# The fecal biomarker ovotransferrin associates with broiler performance under field conditions

**DOI:** 10.1016/j.psj.2023.103011

**Published:** 2023-08-09

**Authors:** Katrien Rysman, Venessa Eeckhaut, Richard Ducatelle, Filip Van Immerseel

**Affiliations:** Livestock gut health team (LiGHT) Ghent, Department of Pathobiology, Pharmacology and Zoological Medicine, Faculty of Veterinary Medicine, Ghent University, Salisburylaan 133, 9820, Merelbeke, Belgium

**Keywords:** broiler, ovotransferrin, fecal biomarker, gut health, field study

## Abstract

Broilers often suffer from subclinical intestinal health problems of ill-defined etiology, which have a negative impact on performance. Macroscopic and microscopic evaluations can be used to monitor intestinal health, but because these are subjective and time-consuming, respectively, objective and easy-to-measure biomarkers are urgently needed. Fecal biomarkers can potentially be used as noninvasive, objective measures to evaluate gut health in broilers. The aim of the current study was to evaluate ovotransferrin (**OVT**) as a biomarker in fecal/colonic samples derived from broilers from 27 industrial farms by investigating associations between OVT, broiler performance and gut histology parameters. Eight chickens per farm were randomly selected, weighed and euthanized on d 28 of the production round. A duodenal section was collected to measure the intestinal villus structure (villus length, crypt depth) and the inflammatory status of the gut (CD3^+^ T-lymphocytes area percentage). The coefficient of variation for the OVT (between farms; 83.45%, within farms; 95.13%) was high compared to the villus length (between farms; 10.91%, within farms; 15.48%), crypt depth (between farms; 15.91%, within farms; 14.10%), villus-to-crypt ratio (between farms; 22.08%, within farms; 20.53%), and CD3^+^ (between farms; 36.38%, within farms; 26.13%). At farm level, colonic OVT was significantly associated with the average slaughter weight (*P* = 0.005), daily weight gain (*P* = 0.007) and the European production index (**EPI**) (*P* = 0.009). At broiler level, significant associations were found between colonic OVT and the villus length (*P* = 0.044) and between the colonic OVT and villus-to-crypt ratio (*P* = 0.050). These results thus show that quantifying OVT in colon can have merit for evaluation of intestinal health in broilers under field conditions.

## INTRODUCTION

In conventional fast growing broiler flocks, performance parameters such as the feed conversion ratio (**FCR**) and daily growth rate are often negatively affected by a dysfunctional digestive system (Timbermont et al., 2011). Common well-known diseases (i.e., coccidiosis or necrotic enteritis), multifactorial enteric syndromes (i.e., dysbiosis) but also environmental triggers, including heat stress, can cause intestinal abnormalities (Marchini et al., 2011; Van Limbergen et al., 2020). Proper monitoring of intestinal health is important but hampered by the lack of good methods under field conditions. Several infectious agents, including *Eimeria* and *Clostridium perfringens,* can cause clinical diseases (coccidiosis and necrotic enteritis) and are easy to diagnose (Goossens et al., 2018). However, a broad array of stressors that involve dietary and environmental challenges can also induce subclinical entities and syndromes which are less easy to diagnose (Timbermont et al., 2011; Van Limbergen et al., 2020). These subclinical entities and syndromes are classified under the term dysbiosis, defined as a multifactorial nonspecific syndrome and characterized by subclinical lesions of gut inflammation and gut barrier disruption, eventually leading to loss of performance and thus economic losses (Bailey, 2010; De Gussem, 2010; Timbermont et al., 2011). Such subclinical diseases (i.e., gut dysbiosis) are currently monitored using macroscopic scoring systems (Teirlynck et al., 2011). However, these current diagnostic methods lack objectivity, are labor-intensive and invasive (De Meyer et al., 2019). Microscopic evaluation of intestinal villus morphology and inflammation is the gold standard but it is labor- and time-consuming, thus highlighting the need for alternative noninvasive diagnostic techniques which allow to detect intestinal damage at an early stage, preferably at flock level (Ducatelle et al., 2018). An easy to measure and objective approach would help to overcome this diagnostic gap enabling to respond quickly by adjusting broiler management and/or feed composition. Also the need for preventive and therapeutic interventions with antibiotics for the control of intestinal diseases can possibly be reduced when better diagnostic methods are available, which could have a positive impact on the problem of antimicrobial resistance spread (Persoons et al., 2012; Ducatelle et al., 2018). Over the past years several research groups searched and identified potential fecal protein biomarkers of intestinal health under experimental settings (Chen et al., 2015; Goossens et al., 2018; De Meyer et al., 2019; Barekatain et al., 2020; Dal Pont et al., 2021). Biomarkers are defined as quantitative objective measurements of biological processes which can change in abundance under pathological circumstances (Strimbu and Tavel, 2010). They can be detected in various biological samples such as serum (Rath et al., 2009) and fecal material (Goossens et al., 2018; De Meyer et al., 2019; Barekatain et al., 2020) and can be indicators of inflammation, gut barrier leakage and/or tissue damage (De Meyer et al., 2019). Ovotransferrin (**OVT**) has been suggested as a candidate protein biomarker for intestinal health evaluation (Rath et al., 2009; Goossens et al., 2018). This glycoprotein is a moderate positive acute phase protein (**APP**) with a broad spectrum of functions (Giansanti et al., 2012). Besides the role of OVT in the innate immune system, it also has antimicrobial activity, is immunomodulating and anti-oxidant (Giansanti et al., 2012; Wu and Acero-Lopez, 2012). This glycoprotein has originally been proposed as a potential serum biomarker for inflammatory diseases in chickens (Rath et al., 2009). More recently it has been identified as a fecal biomarker in an intestinal gut barrier leakage model (Goossens et al., 2018), pointing to leakage of serum proteins and thus increased intestinal barrier permeability. While OVT is well investigated as a serum and fecal biomarker in experimental models, to the best of our knowledge the potential of this biomarker for detection of gut health issues in commercial broilers has not been investigated so far. The objective of the study was therefore to evaluate OVT as a potential fecal biomarker under field conditions, by evaluating associations with broiler performance (individual body weight [at d 28], FCR, average slaughter weight, mortality [%], daily weight gain [g/d], the European production index [**EPI**]) and gut histological parameters (villus length, crypt depth, CD3^+^ T-cell infiltration in the gut wall).

## MATERIALS AND METHODS

### Ethical Statement

Housing and animal care was in accordance with EU Directive 2007/ 43/EC and the Report from the Commission to the European Parliament and the Council on the application of this Directive. Birds were solely euthanized for inspection of the intestinal tract and collection of intestinal samples, and the procedure was done in accordance with Annex I of the Council Regulation (EC) No 1099/2009 of 24 September 2009.

### Study Design and Sampling

Twenty-seven Belgian conventional broiler farms were included in the field study. The broiler farms were visited between 7 August 2019 and 17 June 2021. At d 28 of one production round, 8 randomly selected chickens (Ross 308) were weighed and euthanized by intravenous injection of an overdose of barbiturates. A duodenal segment (5 cm) was collected at the curvature of the duodenal loop and fixed in 4% phosphate buffered formaldehyde. Colon content was collected and immediately stored at -20°C.

### Enzyme‑Linked Immunosorbent Assay for Quantification of OVT

In total, 216 colon samples were randomly selected from 27 conventional broiler farms, which corresponds to 8 samples per farm. Unprocessed colon content samples were thawed, weighted (150 mg) and diluted in 1500 µL TBS (50 mM Tris, 150 mM NaCl, pH = 7.2) with protease inhibitor cocktail (P2714, Sigma-Aldrich). After 2 × 1 min of vortexing, the samples were centrifuged (13.000 × *g*, 10 min, 4°C). The supernatant was transferred to an Eppendorf tube and immediately stored at -20°C. A commercial enzyme‑linked immunosorbent assay (**ELISA**) kit (Chicken Ovotransferrin ELISA, KT-530, Kamiya Biomedical Company, Tukwila, WA) was used to determine the OVT concentrations in the colon samples. The assay protocol of the manufacturer was used with supernatant that was diluted 1:20.

### Morphometrical Evaluation

The duodenal loops were fixed for 24 h, dehydrated in xylene and embedded in paraffin. Sections of 4 µm were cut using a microtome (HM360, Thermo Scientific, Waltham, MA) and processed as described by De Maesschalck et al. (2015). After staining with hematoxylin and eosin, morphologic parameters were assessed using standard light microscopy. Villus length, measured from the crypt–villus junction to the villus tip, and crypt depth, measured from the junction to the base, were determined in the duodenum by random measurement of 10 villi per section at 5× magnification using a Leica DM LB2 microscope equipped with a camera and a computer based image analysis program, LAS V4.1 (Leica Application Suite V4, Wetzlar, Germany).

### Immunohistochemical Examination

Antigen retrieval was performed on 4 µm duodenal sections with a pressure cooker in citrate buffer (10 mM, pH 6). Slides were rinsed with washing buffer (Dako kit, K4011, Glostrup, Denmark) and blocked with peroxidase reagent (Dako, S2023) for 5 min. Slides were rinsed with distilled water and Dako washing buffer before incubation with anti-CD3 primary antibodies (Dako CD3, A0452) for 30 min at room temperature diluted 1:100 in antibody diluent (Dako, S3022). After rinsing again with washing buffer, slides were incubated with labelled polymer-HRP anti-rabbit Ig (Dako Envision+ System-HRP, K4011) for 30 min at room temperature. Before adding di-amino-benzidine (DAB+) substrate and DAB+ chromogen (Dako kit, K4011) for 5 min, slides were rinsed 2 times with washing buffer. To stop the staining, the slides were rinsed with distilled water, dehydrated using the Shandon Varistain-Gemini Automated Slide Stainer and counterstained with hematoxylin for 10 s. The slides were analyzed with a Leica DM LB2 microscope and a computer based image analysis program LAS V4.1 (Leica Application Suite V4) to measure CD3^+^ cells in a total area of 3.5 ± 0.5 mm^2^ which represents the area of approximately 10 to 12 villi.

*Data collection* On broiler level, data included individual body weight and the morphometric and immunohistochemical parameters for each bird individually.

Farm level data included median values of the morphometric and immunohistochemical parameters and the technical results (FCR, average weight at slaughter (kg), daily weight gain (g/d), mortality (%) and the EPI) of 27 commercial broiler farms.

The EPI was calculated using the following formula:

$$\frac{\left(\text{Average}\;\text{body}\;\text{weight}\right)\;\times \;\left(\text{livability}\;\left(100-\text{dropout}\%\right)\right)}{\left(\text{FCR}\right)\;\times \;\left(\text{average}\;\text{age}\;\text{at}\;\text{slaughter}\right)}\;\times \;100$$

### Statistical Analysis

The statistical software R 4.1.3 in R studio (R Core Team, 2021) was used to perform data analysis on farm level.

The descriptives of the histological parameters and OVT concentration is described as follows: mean ± SD (min – max). The variation of the OVT concentration and the histological parameters (between and within farms) was determined with the coefficient of variation (**CV**). Eight birds per farm were used to obtain the CV within farms. The CV between farms was calculated using the mean value and standard deviation of each individual farm.

On farm level, a linear model was used to evaluate the linear relationship between the median values of the histological parameters and OVT concentrations and between the broiler performance and OVT concentrations (R Core Team, 2021). The OVT concentration was included as the fixed effect. The histological parameters (villus length, crypt depth, villus-to-crypt ratio, CD3^+^) and broiler performance parameters (FCR, average weight at slaughter, body weight, daily weight gain, mortality and EPI) were included as the dependent variable for each linear model individually. A result with a *P*-value ≤0.05 was considered statistically significant.

On bird level, linear associations between OVT concentrations and histological parameters were investigated with a linear mixed model (R Core Team, 2021). The OVT concentration was included as the dependent variable and the farm as random effect. Each model contained a histological parameter (villus length, crypt depth, villus-to-crypt ratio, CD3^+^) or the body weight (D28) as fixed effect and the gender was included as an independent variable. A joint.test (i.e., ANOVA type III test) was used to evaluate the result of the multiple linear mixed models (Lenth, 2022).

## RESULTS

### Between and Within Farm Variation of the OVT Concentration

**Figure 1:**
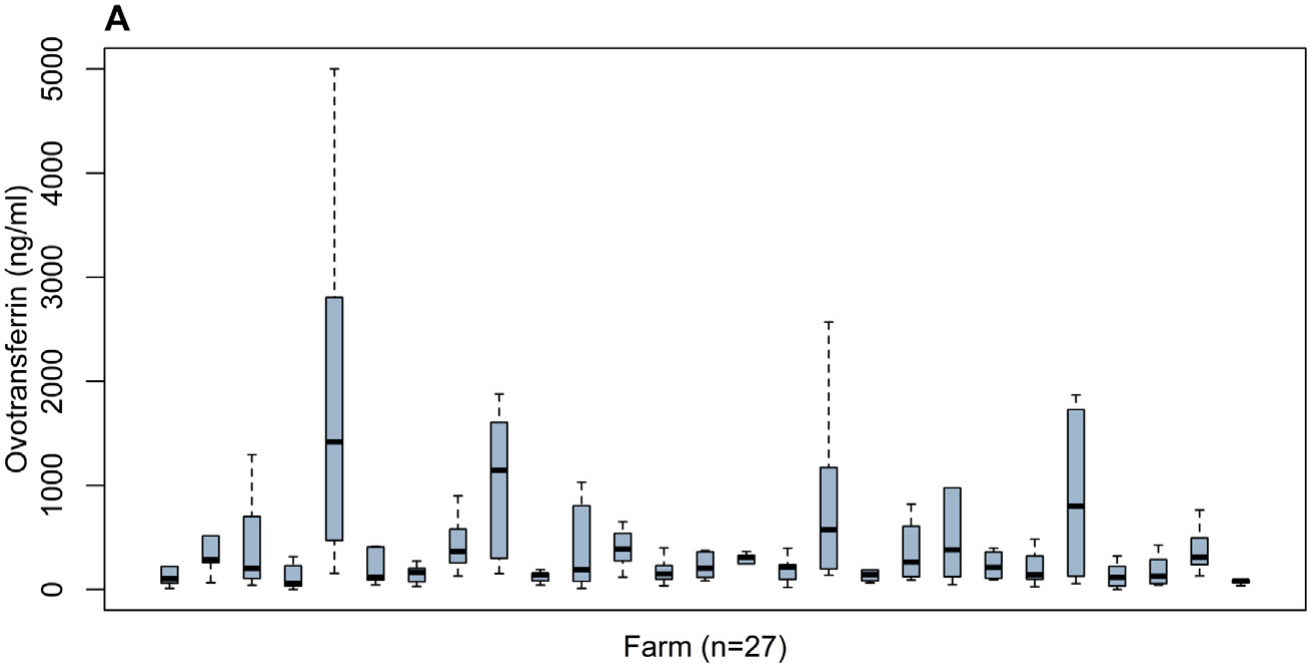
Boxplot illustrating the ovotransferrin concentration (ng/mL) measured in colon content from 8 animals per farm (n = 27).

Table 1 and Figure 1 present the descriptive statistics of the OVT concentrations derived from 8 birds per farm (n = 27). In addition, Table 1 illustrates the variation of the histological parameters (CD3^+^ [%], villus length, crypt depth, villus-to-crypt ratio) and the OVT concentration. The OVT concentration (ng/mL) had a range between 82.56 and 1,573.58 ng/mL with an average of 476.96 ng/mL. The range of the villus length was between 1,111.72 µm and 1,772.86 µm with an average of 1,474.28 µm. The range for the crypt depth was between 240.50 µm and 464.48 µm with an average of 350.65 µm. The mean values were 4.42 ± 0.97 (3.02–6.63) for villus-to-crypt ratio and 11.87 ± 4.32 % (4.47–20.41%) for the T-cell area percentage (CD3^+^). The CV was used to evaluate the variation between and within farms. The CV (%) of the OVT was higher compared to the histological parameters with values of 83.45% between farms and 95.13 % (44.43–167.03%) within farms. Among the histological parameters, the highest CV (%) was found for the CD3^+^(%) with a CV% between farms of 36.38% and a CV (%) within farms of 26.13% (12.02–40.65%). We found the lowest CV (%) for the villus length with values between farms of 10.91% and within farms of 15.48% (6.09–25.62%). The CV (%) between farms was 22.08 % for the villus-to-crypt ratio and (15.91%) for the crypt depth. Within farms, the CV (%) was 20.53% (9.08–38.39%) for the villus-to-crypt ratio and 14.10% (4.07–25.27%) for the crypt depth. The CV (between and within farms) of the histological parameters is in line with the study of Ringenier et al. (2021).

**Table 1:** The variation of the OVT concentrations and histological parameters is expressed with coefficient of variation (CV) between and within farms.

	Mean	SD	Min–max	CV between farms	CV within farms
Villus length (µm)	1474.28	160.85	1111.72–1772.86	10.91%	15.48% (6.09–25.62%)
Crypt depth (µm)	350.65	55.79	240.50–464.48	15.91%	14.10% (4.07–25.27%)
Villus- to- crypt- ratio	4.42	0.97	3.02–6.63	22.08%	20.53% (9.08–38.39%)
CD3^+^ (%)	11.87	4.32	4.47–20.41	36.38%	26.13% (12.02–40.65%)
Ovotransferrin (ng/mL)	476.96	398.02	82.56–1573.58	83.45%	95.13% (44.43–167.03%)

OVT concentrations and histological parameters are derived from 27 broiler farms (n = 216 colon samples).

Abbreviation: OVT, ovotransferrin.

### Associations Between Histological Parameters and OVT on Farm Level

Associations on farm level between the OVT concentration (ng/mL) and histological parameters (CD3^+^ (%), villus length, crypt depth, villus-to-crypt ratio) were examined with a linear model. OVT concentration did not show significant associations with the histological parameters (CD3^+^ [%] *P* = 0.709, villus length *P* = 0.099, crypt depth *P* = 0.376, villus-to-crypt ratio *P* = 0.139).

### Associations Between OVT and Broiler Performance on Farm Level

**Figure 2:**
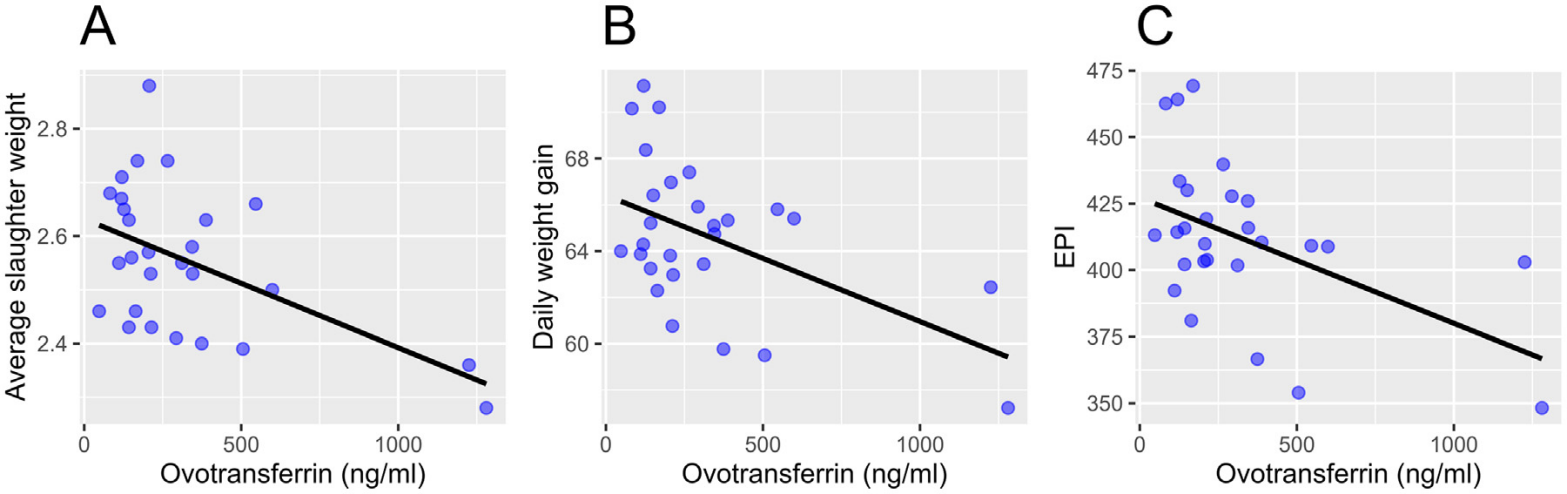
Scatterplots illustrating the significant associations between the OVT concentration and (A) the average slaughter weight, (B) the daily weight gain (g/d), (C) and the EPI of 27 commercial broilers farms. The association between parameters is illustrated with regression lines. Abbreviations: EPI, European production index; OVT, ovotransferrin.

A linear model was used to evaluate associations between OVT and broiler performance (FCR, average weight at slaughter [kg], daily weight gain [g/d], mortality [%], EPI) (Figure 2) on farm level. A significant association was found between the OVT concentration and the average slaughter weight (*P* = 0.005), daily weight gain (*P* = 0.007), and the EPI (*P* = 0.009). No significant association was found between the OVT concentration and the FCR (*P* = 0.122) and between the OVT concentration and mortality (%) (*P* = 0.625).

### Associations Between Histological Parameters and OVT on Broiler Level

**Figure 3:**
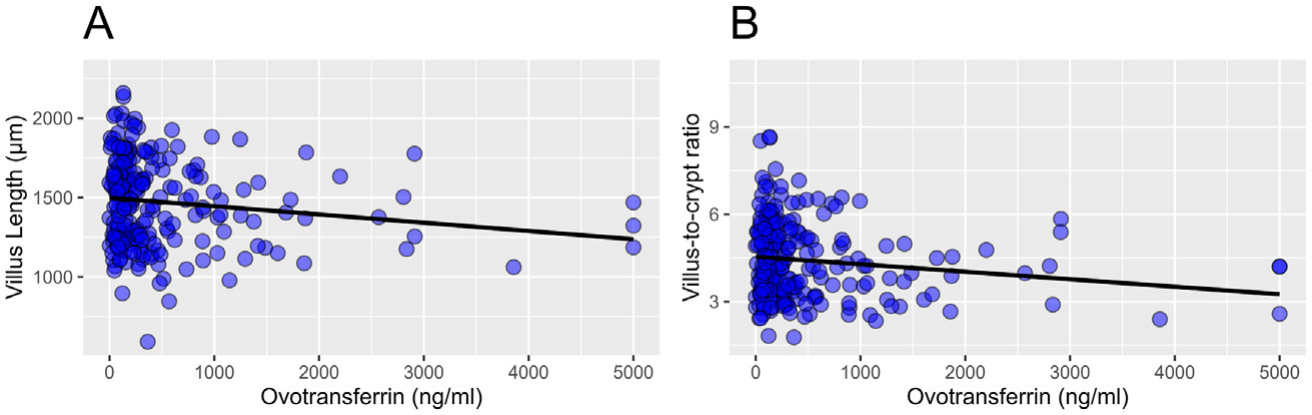
Scatterplots illustrating a significant association between the OVT and the villus length (A) and between the OVT and the villus-to-crypt ratio (B) on individual broiler level. The linear association is presented with a regression line. Abbreviation: OVT, ovotransferrin.

On broiler level, a linear mixed model revealed a significant association between the OVT concentration and the villus length (*P* = 0.044) and a borderline significant association between the OVT concentration and the villus-to-crypt ratio (*P* = 0.050) (Figure 3). The association between the OVT concentration and crypt depth (*P* = 0.342) and between the OVT concentration and CD3^+^ T-cell area percentage (*P* = 0.327) was not statistically significant on broiler level.

## DISCUSSION

The early diagnosis of (sub-) clinical gut health disorders remains difficult in intensive poultry systems. The currently available tools for gut health diagnostics have disadvantages as being invasive, subjective, labor-intensive and/or time consuming (De Meyer et al., 2019) creating an urgent demand for noninvasive objective diagnostic tools which allow to detect intestinal health issues early on in the production round (Goossens et al., 2018; Dal Pont et al., 2021).

Biomarkers are defined as measurable alterations in biological substances that associate with normal or abnormal conditions (Strimbu and Tavel, 2010). They can be detected in a range of different biological samples including blood, cerebrospinal fluid or fecal material (Strimbu and Tavel, 2010). Recently, extensive experimental research has led to the identification of multiple candidate host biomarkers of gut health in serum and feces (Goossens et al., 2018; De Meyer et al., 2019; Barekatain et al., 2020; Dal Pont et al., 2021), but the validation of these biomarkers in field samples has not yet been performed. Studies identifying serum biomarkers to evaluate gut health were already carried out in broiler chickens (Chen et al., 2015; Gilani et al., 2017; Ducatelle et al., 2018). However, many blood biomarkers can also be elevated in case of nonintestinal diseases, making them nonspecific (Rath et al., 2009; Goossens et al., 2018).

A broad range of factors can influence gut health under field conditions making gut health evaluation in the field more complex as compared to experimental modelling approaches with infectious agents that cause clinical pathology. Indeed, under field conditions, the intestinal health of broiler chickens is influenced by factors such as the management system, environmental conditions (as heat stress), enteric pathogens (as *Eimeria* spp, *C. perfringens*), and the feed quality and composition, all of which can help to explain farm level variability (Teirlynck et al., 2011; Goossens et al., 2018; Van Limbergen et al., 2020; Gilani et al., 2021).

To the best of our knowledge, the present study is the first to evaluate the use of a fecal/intestinal biomarker, OVT, under field conditions. This marker was chosen because previous research already demonstrated elevated intestinal and fecal OVT levels in chickens with coccidiosis and necrotic enteritis under experimental conditions (Goossens et al., 2018).

OVT is an APP in chickens (Xie et al., 2002). The acute phase response is a prominent systemic reaction of the organism to local or systemic disturbances in its homeostasis (Gruys et al., 2005). It can be triggered among other things by severe intestinal inflammation. It is characterized by changes in the metabolism of the liver, which results in downregulation of several secreted hepatic proteins, known as negative APPs, while the expression and secretion of other proteins is upregulated, the latter being classified as positive APPs. OVT is one of the typical positive APPs in the chicken. Its concentration is increased in the blood during the acute phase reaction. In case of gut barrier leakage, plasma proteins leak into the intestinal lumen, making intestinal/fecal OVT an interesting candidate biomarker of intestinal barrier failure.

A high variability of the OVT levels within and between farms was found in this field study. OVT concentration may be used to compare the intestinal health status between farms. Also, the broiler level variability within farms can be used as gut health marker, as a wide variation in the intestinal health status between animals within a farm might indicate a problem that is present in a subset of the animals.

There are, however, a number of factors that may complicate the use of OVT ELISA as a simple tool to evaluate intestinal health. Indeed, fecal material is a complex matrix with interfering substances as proteolytic enzymes (Dal Pont et al., 2021). OVT is not heat stable as it rapidly degrades at room temperature (Goossens et al., 2018). Therefore, the accurate collection and transport of fecal samples is of utmost importance. We tried to minimize OVT degradation by immediately storing and transporting the colon samples in a -20°C freezer, and a protease inhibitor cocktail was used during the extraction procedure. This does complicate gut heath diagnostics, as in the field it is much more difficult to logistically organize the proper sampling method.

A good biomarker must comply to a broad range of characteristics as being sensitive, specific, reliable, robust, stable and minimally invasive (Ducatelle et al., 2018; Dal Pont et al., 2021). The biomarker measurements should thus show little or no variability under healthy conditions and should increase in case of gut health alterations (Aronson and Ferner, 2017). As OVT is a gut permeability marker, it might be useful to combine it with other biomarkers, such as inflammation markers. For example, calprotectin may be a useful candidate biomarker to combine with OVT, as it has already been successfully measured in both fecal material and serum of broilers, as indicator of inflammation (Dal Pont et al., 2021).

Colonic OVT concentration at d 28 was significantly negatively associated at farm level with growth parameters such as the average slaughter weight, daily weight gain and EPI, calculated at the end of the production round. In addition, a significant association was found between the gut morphological parameters (villus length, villus-to-crypt ratio) and OVT levels at broiler level. Shortening of villi may indicate increased loss of epithelial cells. These cells are being detached from surrounding cells and from the basement membrane, leaving a temporary gap (Bullen et al., 2006). This may account for the barrier leakage. Shortening of villi leads to a decrease in small intestinal surface area, which in turn is a major cause of malabsorption (Ensari, 2014). Malabsorption will inevitably affect growth rate. Moreover, it has clearly been demonstrated in experimental models that in the intestine, inflammation is associated with increased intestinal epithelial cell death, leading to villus atrophy (Parker et al., 2019).

In conclusion, OVT in feces appears to be a valuable indicator of the key phenomena underlying intestinal health issues in broilers. Considering the associations found at farm level with a number of performance parameters at the end of the production round, OVT quantification might be a valuable tool to predict performance. Therefore, further studies should focus on monitoring OVT at earlier stages of the production round.
